# Ontario Veterinary Medical Society

**Published:** 1893-01

**Authors:** H. D. Martin

**Affiliations:** Secretary


*Ontario Veterinary Medical Society:* The following monthly report of the Ontario Veterinary Medical Society shows the active interest of the students in its proceedings

The annual meeting for the election of officers of the Ontario Veterinary Medical Society was held at the College Hall on Temperance Street, on the evening of Oct. 19th, 1892, with the following result:

President, Prof. Andrew Smith, F.R C.V.S.; Vice-President, C. H. Sweetapple, V. S.; Secretary, H. D. Martin; Assistant Secretary, A. C. Baker; Treasurer, J. Stover; Librarian, G. W. Dunlap.

After which the Society listened to the following papers:

*Essays.* Tetanus, L. G. Moore; Cataract, F. Evans; Periodic Opthalmia, C. J. Sigmond.

*Communications:* Partial Paralysis of Lip, E. D. Block.

*Oct 21, Essays:* Parturition, W. H. Kerr; Heredity, F. Abele; Treatment of Wounds, W. B. Switzer; Influenza, G. W. Dunlap; Function of Kidneys, J. McGillicuddy; Aloes, J. A. Fillsinger.

*Communications:* Azoturia, E D. Longacre; Vovulus, F. Abele.

*Oct. 25, Essays:* Pleuro Pneumonia, L. Van Es; Corns, H. G. Sands; Navivular Disease, E. E. Shoebottom; Amaurosis, O. J. Biehu; Diseases of Bladder, A. E. Alexander.

*Communications:* Æsophogotomy, L. Van Es; Lacerated Tongue, F. Evans; Diarrhœa, W. McBride; Lymphangitis, D. Kohler.

*Nov. 1, Essays:* Influenza, R. Perkins; Prop of foot for Shoe, G. Howell; Tetanus, E. L. Kolb.

*Communications:* Dehorning, G. R. Christian; Strongylus Tetracanthus, J. Airth; Spaying of Cows, H. F. Kennedy; Parturient Apoplexy, Chas. H. Adams; Complications, F. H. Cassel.

*Nov. 4, Essays:* Enteritis, F. M. Blackford; Castration, A. W. McKay; Handling and Nursing, W. H. B. Medd.

*Communications:* Acute Indigestion, J. Halton; Fracture of Metacarpal, J; P. Stover; Rupture of Vagina, A. C. Baker; Metrotomy, C. J. Sigmond; Stomatitis. W. H. B. Medd.

*Nov. 8, Essays:* Diarrhœa, M. H. Kuhl; Tetanus, H. F. Kennedy; Parturient Apoplexy, E. D. Block; Indigestion, M. H. Moore.

*Communications:* Acute Laminitis, W. H. Goddes; Protrusion of Rectum, C. M. Cuthbertson; Mammitis, C. E. Wright; Laceration of the Eye, D. Glendinning.

*Nov 11, Essays:* Azoturia, H. H. Post; Parturient Apoplexy, D. R. Kohler.

*Communications:* Scrotal Hernia, W. B. Washburn; Acute Indigestion, M. H. Moore; Punctured Wound, H. M. Gohn; Carbuncle of Coronary Band, W. F. Coleman.

*Nov. 15, Essays:* Veterinary Dentistry, H. J. Weld; Pneumonia, R. F. Campbell; Springhalt, E. J. Sweeley.

*Communications:* Schirrus Cord, C. E. Parker ; Punctured Abdomen, W. H. Derr ; Sunstroke, E. W. Brumter ; Fracture of Inferior Maxillary, B. R. Ilsley ; Carbuncle of Coronary Band, G. W. Dunlap ; Dislocation of the Patella, A. C. Ewart.

*Nov. 18, Essays:* Roaring, M. Kellam ; Indigestion, J. E. Aleya ; Foreign Substance in Anterior Chamber of Eye, J. L. Brooks.

*Communications:* Curb, J. H. Medd ; Lacerated Wound, M. H. Kuhl ; Descent of Peritoneum, R. J. Jelly, Rectal Abscess, F. E. Freeman.

Yours Respectfully,

H. D. MARTIN, *Secretary.*

